# Geographic factors predict wild food and nonfood NTFP collection by households across four African countries

**DOI:** 10.1016/j.forpol.2018.08.002

**Published:** 2018-11

**Authors:** Matthew Cooper, Alex Zvoleff, Mariano Gonzalez-Roglich, Felly Tusiime, Mark Musumba, Monica Noon, Peter Alele, Madeleine Nyiratuza

**Affiliations:** a Conservation International, 2011 Crystal Drive #500, Arlington, VA 20222, United States; b University of Maryland, Department of Geographical Sciences, 2181 Samuel J. LeFrak Hall, 7251 Preinkert Drive, College Park, MD 20742, United States; c Africa Innovations Institute, Plot 1544, Koire Close, Old Kiira Road, Bukoto, Kampala, Uganda; d University of Florida, Institute of Food and Agricultural Sciences, P.O. Box 110180, Gainesville, FL 32611-0180, United States; e Wildlife Conservation Society, KG 635 St, Kigali, Rwanda

**Keywords:** Non-Timber Forest Products (NTFPs), Wild foods, Forests, Household surveys, Geographic determinants, Africa

## Abstract

Wild foods and other nonfood NTFPs are important for improving food security and supplementing incomes in rural peoples' livelihoods. However, studies on the importance of NTFPs to rural communities are often limited to a few select sites and are conducted in areas that are already known to have high rates of NTFP use. To address this, we examined the role of geographic and household level variables in determining whether a household would report collecting wild foods and other nonfood NTFP across 25 agro-ecological landscapes in Tanzania, Rwanda, Uganda and Ghana. The aim of this study was to contribute to the literature on NTFP collection in Africa and to better understand where people depend on these resources by drawing on a broad range of sites that were highly variable in geographic characteristics as well as rates of NTFP collection to provide a better understanding of the determinants of NTFP collection. We found that geographic factors, such as the presence of forests, non-forest natural areas like grasslands and shrublands, and lower population density significantly predict whether a household will report collecting NTFP, and that these factors have greater explanatory power than household characteristics

## Highlights

• Data was collected from 751 households located across 4 African countries.
• Geographic factors significantly predict whether households collect NTFP.
• Natural land cover types were associated with collecting wild food NTFP.
• Low population densities were associated with nonfood NTFP.

## 1 Introduction

Ecosystem services are critical to human well-being (Haines-Young and Potschin, 2010). Throughout the world, natural and human-impacted areas provide regulating, cultural and provisioning ecosystem services (Bennett et al., 2009), and non-timber forest products (NTFPs) are a provisioning ecosystem service that supports human livelihoods in both developed and developing countries (Shackleton et al., 2015; Sisak et al., 2016; Živojinović et al., 2017). In agrarian parts of the developing world, communities depend significantly on local provisioning ecosystem services for their health and income (Altieri, 2004; Zenteno et al., 2013). While agricultural production often provides the bulk of food and income in these areas, provisioning ecosystem services from forests, shrublands and grasslands also make significant contributions to communities' livelihoods (Ambrose-Oji, 2003; Heubach et al., 2011; Kar and Jacobson, 2012). Understanding the geographic and demographic characteristics of areas that depend on provisioning services in the form of NTFPs is key to conservation strategies that maximize NTFP availability to support human livelihoods and well-being (Angelsen et al., 2011; Kareiva, 2011).

It has been estimated that NTFPs provide income and nutrition for over two-thirds of Africa's population (CIFOR, 2005). These products provide significant income to households and communities, with some products like shea oil and gum arabic being collected and exported to international markets (Mujawamariya and Karimov, 2014; Rousseau et al., 2017). Many other products, such as fuelwood and building materials, are also sold locally and are an income source. A global literature review of 51 case studies across 17 developing countries estimated that, on average, forests provide 22% of a household's total income (Vedeld et al., 2007). While access to NTFPs is often moderated by political and cultural institutions (Lambini and Nguyen, 2014; Ludvig et al., 2016), a common feature of NTFPs is that they do not require financial capital to procure. Thus, households with less income tend to be the most dependent on forest products for food, fuel and materials (Vedeld et al., 2007).

In addition to providing income and supplying goods that households would otherwise have to purchase from markets, NTFPs also support nutrition outcomes, and many wild foods are consumed directly by the household that collected them. Given that forests and other natural areas offer significantly more species for consumption than agriculture alone, wild foods can significantly increase a household's dietary diversity (Powell et al., 2015; Remans and Smukler, 2013) and also provide an income source (Ingram et al., 2017). A study in Madagascar found that removing households' access to wildlife for consumption would increase rates of child anemia by 29% due to decreased meat consumption (Golden et al., 2011). While some wild foods are consumed continuously, many others are a reserve food supply used during times of famine. These “famine foods” are not preferred but are essential for households during hungry seasons or years when agricultural output is low (Mavengahama et al., 2013). Such foods increase household resilience to climate shocks. In surveys of households' climate adaptation strategies in Mali, Tanzania, and Zambia, forests were found to play a key role in reducing vulnerability during droughts and floods by providing alternative food and income sources (Robledo et al., 2012).

While forests are significant providers of NTFP and provisioning ecosystem services, products sourced from other natural areas like shrublands and grasslands also play a significant role in households' livelihoods (Pouliot and Treue, 2013). Because access to forested land is sometimes more regulated than access to grassland and shrubland, these non-forested areas can be a significant resource to less well-connected or less wealthy rural people, such as women or ethnic minorities (Pouliot and Treue, 2013). Whether products sourced from these areas can be included in the term “NTFP” is debatable, as a NTFP can often refer to many types of products sourced from a wide variety of environmental areas and land cover types (Belcher, 2003). For example, some trees that provide products typically classified as NTFPs, such as the Gum Arabic tree (*Senegalia senegal*), often grow in areas with less than the 10% canopy cover required to meet the FAO definition of a forest (FAO, 2012). Furthermore, products sourced from uncultivated non-forest areas have the basic fundamental economic characteristics of NTFPs identified in a comprehensive paper from the Center for International Forestry Research (CIFOR) on NTFPs and rural livelihoods: (i) they have low returns per unit area; (ii) they are primarily used for subsistence and often fill income gaps; and (iii) they are not planted, and are only managed indirectly, if at all (Angelsen and Wunder, 2003). Thus, while this paper examines foods from both forested and non-forested areas like grasslands and shrublands, we use the term NTFP to refer to provisioning ecosystem services sourced from any natural area following the characterization laid out by CIFOR (Angelsen and Wunder, 2003). In our analyses, we split NTFP into two categories: “wild foods” for NTFP like nuts, seeds, bushmeat, honey, or insects, and “nonfood NTFP” for other products such as building materials, medicines, and fibers. When speaking about both wild foods and nonfood NTFP, we use the general term NTFP.

While the benefit that NTFPs provide in supporting rural livelihoods has been clearly demonstrated in many case studies, few studies have been conducted at national and multinational scales relevant to policymakers or conservation and development practitioners (Reed et al., 2016). Indeed, a recent literature review lamented that this body of work is “limited by the propensity for small-scale and short-term evaluations” (Reed et al., 2016). Some notable exceptions to the preponderance of case studies include literature reviews on topics like wild food consumption (Powell et al., 2015) and environmental income from forests (Vedeld et al., 2007), as well as the Population-Environment Network (PEN) dataset on household NTFP use based on surveys conducted in 24 developing countries (Angelsen et al., 2014; Hickey et al., 2016). While these literature reviews and the PEN study have made significant contributions to our understanding of characteristics of households that depend on NTFPs and the degree of their dependence, they have a significant sampling bias, with most of the case studies and sample sites established opportunistically in areas with significant forest cover and where communities were already known to utilize forest resources. Thus, findings from these studies showing that NTFPs provide 22% of total income (Vedeld et al., 2007) or 28% of total income (Angelsen et al., 2014) cannot be taken as representative of all rural developing countries or as representative of any one country.

The fact that studies of household use of NTFPs are usually only conducted in highly localized case studies is unfortunate, as a growing body of literature is beginning to associate various environmental data metrics from satellite imagery with indicators of income, health, and food security from household surveys. Such research has found relationships between an increased Normalized Difference Vegetation Index (NDVI) and decreased child mortality (Brown et al., 2014); more forest cover and greater dietary diversity (Ickowitz et al., 2014); and more forest cover and decreased child stunting (Johnson et al., 2013). Many of these studies have found significant associations, but the specific mechanisms underlying linkages between environmental indicators like NDVI and forest cover with human well-being remain under-explored at relevant scales. This is largely because multinational surveys on human well-being, such as Demographic and Health Surveys (DHS) and Living Standards Measurement Surveys (LSMS), do not collect data on the accessibility and collection of wild foods and non-food products in a standardized manner across countries. On the other hand, datasets that do include data on NTFP use, such as individual case studies or the PEN dataset, do not include detailed data on key measures of human well-being, such as agricultural production, health, and food security. Thus, datasets that can be used to find a significant relationship between vegetation indices or land cover and human well-being at multinational scales are often lacking data on the exact causal linkages. For example, a recent study showed that forest cover was associated with dietary diversity across 21 African countries (Ickowitz et al., 2014, p. 290), but could not explain the exact linkages, stating:

“while we have found clear evidence linking tree cover and indicators of diet quality, we are not able to determine the drivers of this relationship. Our data do not allow us to distinguish between natural forests, old fallows, and agro-forests; thus we cannot ascertain if people living near forests are collecting more nutritious foods from the forest or if they are cultivating them on farms and in agroforests, or a combination.”

This paper aims to bridge these gaps – to provide a characterization of households that gather both food and nonfood NTFP in terms of both household characteristics and environmental characteristics. We do this by examining which geographical and household level variables are significant predictors of household wild food and nonfood gathering from 25 agro-ecological landscapes in 4 countries. While the landscapes in this study were not selected at random, they were selected purposively to monitor a variety of topics such as agricultural intensification, livelihoods, and environmental quality. Thus, landscapes were not selected with the specific intention of examining wild food or NTFP collection, and some of the landscapes selected had no households that reported collecting any NTFPs. This dataset therefore provides a unique opportunity to examine variation in NTFP gathering across and within multiple African countries and agro-ecological regions, as well as the factors associated with that variation, without relying on sample data that was collected in areas already known to have high levels of NTFP gathering. A geographic characterization of households that collect NTFP can, in turn, begin to fill in gaps in knowledge of the mechanisms by which ecosystem provisioning services (measured by satellite-derived environmental indices) could be contributing to positive human health outcomes. Finally, an understanding of which landscapes contain households that collect NTFP in significant numbers can aid conservation priority setting efforts that aim to maximize ecosystem service provision.

## 2 Methods and data

**Fig. 1:**
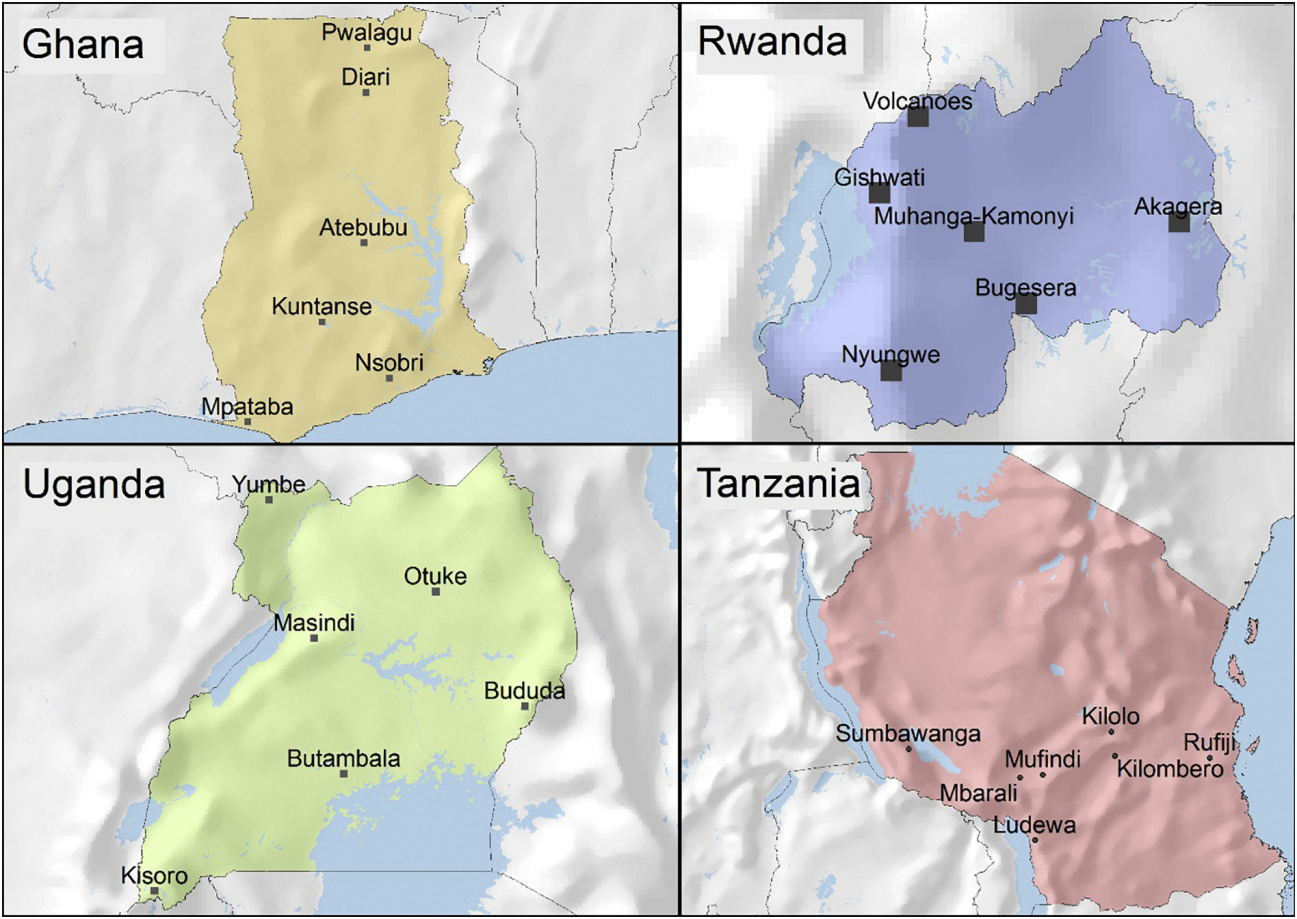
Location of landscapes within the four Vital Signs countries. Each landscape is 10 x 10 km.

For household survey data, we used data from the Vital Signs project (Scholes et al., 2013). Vital Signs is an integrated monitoring system that collects data on agriculture, the environment and livelihoods in a number of agricultural landscapes in Africa. The sampling design involves six to seven 10 × 10 km agricultural landscapes per country, with about 30 households per landscape. Landscapes were purposively placed within the identified regions in each country with the intention to cover a wide distribution of agro-ecological zones in areas where smallholder agriculture predominates (Scholes et al., 2013). Each household was interviewed about agricultural practices and production, off-farm and on-farm income, food security, and collection of food and nonfood NTFPs. A total of 751 households were interviewed across 25 landscapes in Ghana, Uganda, Rwanda and southern Tanzania (See Fig. 1). Data was collected from 2013 to 2016, with interview dates varying by landscape and country. The median amount of time spent in a landscape conducting household surveys was 20 days.

This study used multilevel logit models to determine the most significant geographic and household predictors of whether a household reported collecting NTFPs. Two separate regressions were run: one for whether the household collected wild foods and one for whether the household collected any nonfood NTFPs. The regressions were based on 751 households from Ghana, Uganda, Rwanda, and southern Tanzania.

While many analyses of wild foods include all undomesticated species, including those sourced from farmlands and villages (Powell et al., 2015), the Vital Signs questionnaire specifically asked about wild foods and other nonfood products collected from “nearby fallow lands, forest, woodland, shrubland, rivers, creeks, or other areas.” Households were specifically asked about wild meat, wild insects, fish from local rivers/creeks, nuts or seeds, honey, building materials and medicinal plants but were also given the option to specify other NTFPs. Other products specified were snails, crabs, mushrooms, green vegetables, sisal, and palms for making mats. Because the particular NTFPs that households collected varied widely from one area to another, regressions were not run for each individual product. We used the same predictor variables for both regressions and allowed intercepts to vary at the landscape level and the country level. Additionally, although ancillary data was collected on frequency of collection and market value of NTFPs, the questionnaires were not designed to allow accurate estimation of values or quantities of all food products. To avoid the possibility of erroneous comparisons between areas, we only used simple binary outcomes.

### 2.1 Household survey data

Household-level data used in the regressions included measures of food security and household wealth, as well as demographic characteristics that have been shown in the literature to be significant predictors of wild product use, including the gender of the household head, average household age, household size, and education as measured by the percent of the household that could read in any language and the average years of schooling for all household members (Coulibaly-Lingani et al., 2009). All household-level data was collected using the Vital Signs household survey questionnaire (Scholes et al., 2013).

As a measure of household food security, an adjusted version of the Household Food Insecurity and Access Scale (HFIAS) was used (Coates et al., 2007). This consisted of eight different coping strategies that a household might have to take in response to food insecurity, such as skipping meals or limiting the variety of food eaten. The scale was calculated as the total number of days in the past week the household had to undertake a given coping strategy, summed across all eight coping strategies. In addition to the HFIAS, because food security does not just consist of food access, availability, and utilization, but also requires temporal stability (Wheeler and von Braun, 2013), we added a temporal aspect with a binary variable of whether the household reported not having enough food to feed the household at any point in the previous year.

For measures of household economic status, we included household income from non-agricultural sources, such as off-farm wage labor and running a household business; the total cost of all expenditures made in the previous year by a household for both food and nonfood products; and the total estimated value of all agricultural products produced in the previous year by a household, estimated as the summed production value of field crops, permanent crops, crop byproducts, crop residue, livestock, and livestock byproducts. Monetary estimates were calculated in local currencies for each country, and then converted to 2015 US dollars.

### 2.2 Household-level geographic data

Because not all of the households fell perfectly within the 10 × 10 km landscape in which they were intended to be sampled, and because there was significant within-landscape variation in land cover types, land cover was measured as a household-level variable. Land cover and protected area data was summarized within a given distance of a household. Regression results for land cover within 7.5 km of a household are included in the body of this paper. However, because the distance people travel to collect resources can vary significantly based on the resource and location (Maukonen et al., 2014) regression results within 2.5 km, 5 km, 10 km, and 15 km are included in Appendix A.

Two variables were generated at the household level as indicators of the prevalence of land cover types that might provide wild foods and nonfood NTFPs: one for area covered by only forest and another for area covered by any non-forest, non-agricultural land cover types. Land cover data came from the 300 m spatial resolution European Space Agency Climate Change Initiative (ESA CCI) land cover dataset (Defourny et al., 2017). Forest categories consisted of any land cover type with >15% tree cover, including broadleaved, needleleaved, evergreen, deciduous, and flooded areas, while non-forest, non-agricultural categories (henceforth referred to as “grassland”) consisted of shrubland, grassland, herbaceous and sparsely vegetated areas with <15% tree cover. Because the ESA CCI dataset has annualized data, land cover was extracted for each household for the year in which the survey was conducted.

Additionally, data on protected areas was collected from the World Database of Protected Areas (UNEP-WCMC and IUCN, 2017) and all areas within protected areas (PAs) with International Union for the Conservation of Nature (IUCN) categories I through V were counted as protected, while areas permitting sustainable resource use (category VI) or areas unclassified within the IUCN system were not counted as protected. The variable was calculated as the percentage of total area protected within a given distance of a household. Finally, the 12-month Standardized Precipitation Index (SPI) (Mckee et al., 1993) was calculated for each household at the landscape centerpoint using the 1 km spatial resolution CHIRPS dataset (Funk et al., 2015). The SPI was originally developed to allow inter-comparison of drought and wet periods between stations. The 12-month SPI compares the precipitation total for each set of 12 months to all other 12-month periods in the record. The value of the 12-month SPI in a given month is equal to the number of standard deviations above or below the mean of the total precipitation received in the 12 preceding months (Guttman, 1999). Because households were not all interviewed within the same month, two households in the same landscape could have different SPI values.

### 2.3 Landscape-level geographic data

For each of the 25 landscapes, data on distance to cities and population density were extracted using Google Earth Engine. These factors were selected because they could have an impact on household use of NTFPs, and they were measured at the landscape level because they do not vary significantly over a distance of 10 km. Market distance was counted as the travel time in hours to the nearest town with a population greater than fifty thousand people, and was sourced from the Harvest Choice Market Distance dataset (Harvest Choice, 2011). Population density was measured as the total number of people within each 10 × 10 km landscape in the year 2015, as measured in the 100 km resolution WorldPop dataset (Tatem, 2017).

### 2.4 Variable definitions

Although we used multiple indices of household food security and household income, none of these variables used were found to be multicollinear; however, other potential indices were excluded because of multicollinearity with the indices that we did use. The regression was run in R using the lme4 package version 1.1.12 (Bates et al., 2015) and significance estimates were generated using the lmerTest package version 2.0.32, which uses Satterthwaite's degrees of freedom method to generate significance estimates (Kuznetsova et al., 2014). Variables were rescaled and centered to yield values from −1 to 1 to facilitate model estimation. For a description of each variable, see Table 1.

**Table 1:** Description of variables used in regressions.

Variable	Source	Description
Household survey data		
Head Gender	Vital Signs Survey (Scholes et al., 2013)	Whether the head of household, defined as the household member who occupies the role of decision maker, is male.
Age	Vital Signs Survey	The average age of all household members.
Years of Schooling	Vital Signs Survey	The average years of schooling for household members over 5 years old.
Literacy	Vital Signs Survey	The percentage of individuals over 5 years old who can read in any language.
Household Size	Vital Signs Survey	The number of individuals in the household.
Critical Food Shortage	Vital Signs Survey	Whether the household was unable to meet their basic dietary needs at any point within the past year.
HFIAS	Vital Signs Survey	Household Food Insecurity and Access Score
Total Ag Production	Vital Signs Survey	The total value of all agricultural products produced in the past year, including field crops, permanent crops, crop byproducts, livestock and livestock byproducts in 2015 US dollars
Net Business Income	Vital Signs Survey	The net income from any business run by the household from the previous year in 2015 US dollars
Wage Income	Vital Signs Survey	The total income from wage labor conducted by members of the household over the past year in 2015 US dollars
Nonfood Spending	Vital Signs Survey	The total amount spent on nonfood items over the previous year in 2015 US dollars
Food Spending	Vital Signs Survey	The total amount spent on food over the previous year in 2015 US dollars

Household-level geographic data		
Area Protected	WDPA (UNEP-WCMC and IUCN, 2017)	The percentage of land area within a given distance from a household that falls inside of a protected area.
Forest Cover	ESA-CCI (Defourny et al., 2017)	The percentage of land area within a given distance from a household that is of a forest land cover type.
Grassland	ESA-CCI	The percentage of land area within a given distance from a household that is of a grass, shrub, or herbaceaous land cover type.
12 – month SPI	CHIRPS (Funk et al., 2015)	The Standardized Precipitation Index (SPI) for the 12 months before a survey was conducted.

Landscape-level geographic data		
Market Distance (Landscape Level)	Travel Time to Market Centers (Harvest Choice, 2011)	The number of hours it would take to travel to a town with over 50,000 people from the center of a landscape.
Population Density (Landscape Leve)	WorldPop (Tatem, 2017)	The total population of the 10 km × 10 km landscape from which the households were selected.

## 3 Results

The households in the dataset had significant variation in income, agricultural production, forest cover, and rates of NTFP collection. For example, in Mpataba, Ghana the average agricultural production value per household was $5994 over the previous year, while it was only $286 in Kisoro, Uganda. Similarly, forest cover within 7.5 km of a household ranged from 0.004% in Nsobri, Ghana to 92.7% in Atebubu, Ghana, and rates of NTFP gathering ranged from 0% in Nyungwe and Volcanoes, Rwanda to 87% in Yumbe, Uganda. Finally, the landscapes were placed in areas with ample variation in precipitation, from 861 mm/yr in Sumbawanga, Tanzania to 1618 mm/yr in Mpataba, Ghana. For detailed summary statistics by country and by landscape, including dates of data collection, see Appendix B.

### 3.1 Types and rates of NTFP collecting

Our surveys find wide variability in the rates of collecting wild foods and nonfood NTFPs. The most common NTFP collected was building materials, followed by medicinal plants, while the most common wild food collected was nuts or seeds, followed closely by wild meat (See Tables 2 and 3).

**Table 2:** Number and percentage of households that collected specific wild foods.

Product	Number of Households	Percentage of Households
Nuts or seeds	57	7.6%
Wild meat	54	7.2%
Honey	41	5.5%
Wild insects	18	2.4%
Fish from local rivers/creeks	13	1.7%
Other - Vegetables	7	0.9%
Other - Mushrooms	5	0.7%
Other - Snails	3	0.4%
Other - Crabs	3	0.4%
Any Wild Food	126	16.9%

**Table 3:** Number and percentage of households that collected specific nonfood NTFPs.

Product	Number of Households	Percentage of Households
Building Materials	209	27.8%
Medicinal Plants	170	22.6%
Palms for Mats	2	0.26%
Sisal	1	0.13%
Any Nonfood NTFP	284	37.9%

In looking at the rates of households collecting only wild foods, only nonfood NTFPs, both types of NTFP, or neither wild food nor nonfood NTFPs, over half of households reported collecting no NTFP at all. Additionally, many more households collected nonfood NTFPs than wild foods (See Table 4). A more detailed tabulation is available in Appendix B.

**Table 4:** Tabulation of households that collected only wild foods, only nonfood NTFPs, both wild foods and nonfood NTFPs, or no NTFP at all.

	Number of households	Percentage of households
No NTFPs At All	426	56.6%
Only Nonfood NTFPs	200	26.6%
Only Wild Foods	42	5.6%
Both Wild Food	84	11.2%

### 3.2 Regression results

Across the 25 landscapes, the most significant predictors of whether a household would report collecting wild foods were the presence of forests or grasslands. Household characteristics like demographics, education, income, spending, and food security had little significance in determining whether a household would report collecting wild foods when geographic variables were included in the regressions (See Table 5).

**Table 5:** Predictors of whether a household reported collecting wild food NTFP. Note: variables were centered and rescaled. n = 751. A p-value of <0.001 is indicated with three stars (***), a p-value of <0.01 is indicated with two stars (**), a p-value of <0.05 is indicated with one star (*), and a p-value of <0.1 is indicated with a period (.).

	Estimate	Std. Error	z value	Pr(>|z|)
(Intercept)	−3.40289	1.240898	−2.74228	0.006101**
Head Gender	0.594308	0.448286	1.325734	0.184928
Age	−0.44911	0.895157	−0.50171	0.615873
Years of Schooling	−1.61346	1.132877	−1.42421	0.154385
Literacy	0.068992	0.917154	0.075224	0.940036
Household Size	−0.05441	0.749899	−0.07256	0.942156
Critical Food Shortage	−0.03797	0.345719	−0.10982	0.912553
HFIAS	0.643374	1.376622	0.467357	0.640245
Total Ag Production	0.256779	1.76156	0.145768	0.884105
Net Business Income	−0.00705	1.404397	−0.00502	0.995997
Wage Income	−2.76351	2.256545	−1.22466	0.220702
Nonfood Spending	−0.60565	1.880145	−0.32213	0.747354
Food Spending	−0.51856	0.994423	−0.52147	0.602038
Area Protected	−1.26698	1.458995	−0.86839	0.385178
12 – month SPI	0.067067	0.489361	0.137051	0.89099
Forest Cover	2.025117	0.948489	2.135099	0.032753*
Grassland	2.701474	1.192099	2.266148	0.023442*
Market Distance	0.194492	2.350343	0.08275	0.93405
Population Density	1.177869	1.341293	0.878159	0.379857

Similar to wild foods, household characteristics had little significance for whether a household would report collecting nonfood NTFP. Unlike wild foods, however, land cover (forest cover or grassland) was not a significant predictor. Rather, the best predictor of whether a household would report collecting nonfood NTFP across the 25 landscapes and four countries was lower population density. Additionally, lower household literacy rates and higher HFIAS scores were both somewhat associated with nonfood NTFP collection (See Table 6).

**Table 6:** Predictors of whether a household reported collecting nonfood NTFP. Note: variables were centered and rescaled. n = 751. A p-value of <0.001 is indicated with three stars (***), a p-value of <0.01 is indicated with two stars (**), a p-value of <0.05 is indicated with one star (*), and a p-value of <0.1 is indicated with a period (.).

	Estimate	Std. Error	z value	Pr(>|z|)
(Intercept)	−1.87005	1.090519	−1.71483	0.086377 .
Head Gender	0.347227	0.289995	1.197354	0.231168
Age	−0.39671	0.722252	−0.54927	0.582817
Years of Schooling	0.167523	0.825888	0.20284	0.83926
Literacy	−1.22975	0.737629	−1.66717	0.095481 .
Household Size	−0.34939	0.517634	−0.67498	0.499688
Critical Food Shortage	0.366043	0.245106	1.493406	0.135331
HFIAS	1.805542	0.987053	1.829224	0.067366 .
Total Ag Production	2.075537	1.479594	1.402774	0.160684
Net Business Income	1.355725	1.445619	0.937817	0.348339
Wage Income	1.914139	1.693412	1.130345	0.258331
Nonfood Spending	0.943964	1.380575	0.683747	0.494135
Food Spending	−0.0562	0.902869	−0.06225	0.950366
Area Protected	−1.19388	0.988749	−1.20747	0.227252
12 – month SPI	−0.0928	0.432367	−0.21462	0.830061
Forest Cover	−0.67606	0.966266	−0.69966	0.48414
Grassland	0.406197	1.065113	0.381366	0.702932
Market Distance	−1.24709	1.419427	−0.87858	0.379627
Population Density	−3.08889	1.42321	−2.17037	0.029979*

Regressions were also run at 2.5 km, 5 km, 10 km, and 15 km spatial scales, and these results were included in Appendix A. Many of the variables that were significant predictors at a 7.5 km scale remained significant at all scales. Lower population densities remained a significant predictor of nonfood NTFP collection, even as forest cover, grassland area, and area protected were measured at different scales. For wild food collection, forests were a significant predictor of NTFP collection at all spatial scales and increased in significance at smaller scales. Grassland was most significant at 7.5 and 10 km scales, but lost significance at both larger and smaller scales. Additionally, a lower percentage of area protected was somewhat significant as a predictor of wild food collection at 5 km scales and was significant as a predictor of nonfood NTFP collection at 10 and 15 km scales.

## 4 Discussion

One of the most striking results in this analysis is that geographic variables like land cover and population density are better predictors of whether a household will report collecting NTFP than any household level variables that have been shown to be related to wild product gathering in other contexts (Bakkegaard et al., 2017; Coulibaly-Lingani et al., 2009; Melaku et al., 2014). These findings are in line with a similar study conducted in China, which found that geographic factors like soil quality and forest distance were significant predictors of whether a household would collect NTFP, while household socio-economic factors, such as annual per capital income or education levels, were not (Zhu et al., 2017). The presence of both forests and grasslands were significant predictors of whether a household would report collecting wild foods, while lower population density was significantly associated with higher collection of nonfood NTFPs. Given that there is also substantial variability between landscapes in terms of socio-economic characterization (see Appendix B), it is also apparent that the geographic context, rather than socio-economic factors, is the greatest determinant of whether households in that landscape will report gathering NTFP.

Interestingly, very different contexts determine whether a household will report collecting wild foods or nonfood NTFPs. The fact that environmental land cover types predicted whether a household will report collecting wild food suggest that this land cover variable is likely capturing availability of wild foods in particular land cover types. Both wild meats and wild nuts and seeds, the two most frequently reported types of wild food collected, require some amount of natural habitat in order to grow, and thus are unavailable in areas without these land cover types. Building materials, on the other hand, can often consist of mud bricks or other products that don't necessarily require the presence of a particular land cover type. Even organic building materials, like thatch and wood, can be sourced from marginal areas or small plots, whereas food species of wild meat and plants like shea (*Vitellaria paradoxa*), locust bean (*Parkia biglobosa*), and *Syzygium* fruits require some natural habitat (Naughton et al., 2015). The fact that lower population densities were associated with greater collection of nonfood NTFPs could be duo to a number of factors. It possible that in densely populated areas artificial building materials and medicines are more readily available, that households have higher incomes in densely populated areas to purchase these resources, that there is greater competition for natural building materials and medicines in these areas, or that NTFP availability is quickly exhausted in densely populated areas.

Another significant finding was that household level variables related to demographics, education, food security, and income had little predictive power in determining whether a household would report collecting NTFPs. This stands in opposition to pre-existing work on household determinants, which has found that factors like age, household size, education levels, and income sources are significant determinants of whether a household would report having access to NTFPs (Coulibaly-Lingani et al., 2009). Where our models did find that household level predictors were somewhat significant, they concurred with previous literature: both decreased household literacy and decreased food security were somewhat associated with greater collection of nonfood NTFPs. This is likely because illiteracy and food insecurity are associated with poorer and marginalized members of communities, which previous studies have found to be more likely to depend on NTFPs (Pouliot and Treue, 2013). It is possible that household-level variables do have significant effects within a landscape, as prior research suggests, but that our sample size was not large enough to detect these relationships. Coulibaly-Lingani sampled over 1800 households in one province of Burkina Faso, and showed that within this small area many household characteristics were significant predictors of NTFP access (Bakkegaard et al., 2017; Coulibaly-Lingani et al., 2009). However, when comparing between countries and agro-ecological zones, as the Vital Signs dataset does, it seems that land cover and population density have more explanatory power than household characteristics when determining if NTFP gathering is part of a given household's livelihood strategy. Thus, these geographic and land cover variables should be taken into account in future econometric work on NTFP access and utilization.

Assessing the presence of forests, grasslands and protected areas within varying distances (see Appendix A) also revealed interesting results. The percent of the land covered by forest was most significant as a predictor of wild food collection at very local scales, around 2.5 km, while the percent of land covered by forest within 10 and 15 km of a household had a less significant effect. Grassland was only significant at 7.5 and 10 km scales. Interestingly, the presence of protected areas was also significant at some scales for both wild foods and nonfood NTFP, with a greater presence of protected areas associated with less NTFP gathering. This could be due to a variety of factors, such as exclusion of households from access to NTFPs within protected areas to greater competition for the NTFPs that fall outside of PAs. It could also be due to respondent bias, with households being reluctant to admit to behavior that is illegal or that may appear illegal. Nevertheless, our findings at multiple scales do suggest that PAs have an effect on household's reported NTFP gathering, although not as salient of an effect as the presence of forests and grasslands. This has significant implications for conservation policy, suggesting that restrictive protected areas, such as those with IUCN categories I through IV, may decrease local peoples access to wild foods and nonfood NTFPs. Thus, more research is needed on policy strategies that allow people to maintain their livelihoods while also meeting conservation goals, such as community-based forest management and protected areas permitting sustainable use of resources (Ellis and Porter-Bolland, 2008).

While greater presence of forests and grasslands is significantly associated with wild food collection and low population densities are associated with nonfood NTFP collection, there are many areas in Africa with high population densities where agricultural land use is predominant. In these areas households likely do not collect NTFP, not only because forests and grasslands are less common, but also because they are well protected or highly fragmented and not as productive of wild food species. This is especially true in Rwanda and southwest Uganda, where the Vital Signs data indicates very little wild food or nonfood NTFP collection and there is little substantial natural land cover outside of national parks like Nyungwe and Volcanoes in Rwanda or Bwindi Impenetrable forest in Uganda. Thus, our results show there may be significant populations of smallholder farmers in Africa that rely on little to no NTFP resources. This suggests that the contribution of NTFP to local incomes across all rural households in sub-Saharan Africa may be much lower than the 22% calculated by Vedeld in a literature review or the 28% calculated by the PEN study (Angelsen et al., 2014; Vedeld et al., 2007). At the very least, our data and analyses suggest that NTFP dependence varies widely across different parts of the continent.

One benefit of this study was its multinational approach, providing significant variety in landscape characterization in terms of factors like landcover type, market distance, and population density. This allows us to build on previous studies that have mostly taken place in one country or setting and compare between landscapes and countries to determine which geographical contexts are most associated with households that collect NTFPs. The multilevel models used in this study take advantage of the multinational approach to allow estimates in one country to borrow strength from the other countries in the analysis. Conducting an analysis at this scale also allows us to speak to previous studies conducted at similar scales finding associations between natural landcover and positive human well-being outcomes (Ickowitz et al., 2014; Johnson and Brown, 2014).

Furthermore, increasing food security and access to provisioning ecosystem services is an increasing goal of conservation in developing countries (Shackleton et al., 2015; Tscharntke et al., 2012), and this research can justify conservation schemes designed to increase availability of provisioning ecosystem services to communities, even in areas where case studies of NTFP collection have not been conducted. Nevertheless, there are some risks to missing important local variables when creating multinational statistical models. While we did not have data on cultural diversity, for example, we did allow for intercepts in the model to vary at the landscape scale and the nation scale, with the intent to account for variation in community and national factors among landscapes and countries.

This study had some limitations that must be noted. One issue is that while the landscape locations were not sampled in a way that targets communities that are known to collect NTFP, they were also not randomly sampled, and therefore may exhibit some bias in the representativeness of the households interviewed. Another limitation was that while this survey asked respondents if they collected NTFPs and what kind they collected, it did not explore questions of frequency, uses, and domestication status of NTFP that were collected, as previous work has done (Casas et al., 2007; Heubach et al., 2011; Kar and Jacobson, 2012). Future work could build on our findings to explore factors like how distance to natural land cover relates to NTFP outcomes, how geographic factors affect outcomes such as the frequency of collection of NTFPs or the market value of NTFPs, as well as how different land cover types correspond to the types of NTFPs collected. Such initiatives should increase the sample size to provide a reliable estimate of household characteristics that are related to NTFP collection, and how these characteristics are affected by geographic factors. Additionally, future work could provide more detailed analyses of how the presence of protected areas and the severity of their restrictions affect households' propensity to collect various types of NTFPs. A final limitation in the data is that it is a cross section that does not allow us to examine interannual variability. Collection of data with higher frequency is recommended to control for heterogeneity among households as well as to examine trends in the supply of NTFPs in a given region.

Overall, our findings suggest that the presence of forests and grasslands are significant predictors of whether a household will report collecting wild foods, that a greater presence of these areas leads to a greater likelihood that a household will collect wild foods, and that these geographic variables in fact play a more significant role than a household's income levels or food security status. This is especially true in the four countries where Vital Signs collected data but also likely true for households in areas with similar agro-ecological systems in sub Saharan Africa. These findings are relevant to recent literature associating forest cover with positive outcomes in terms of dietary diversity and child nutrition (Ickowitz et al., 2014; Johnson et al., 2013), suggesting that the collection of wild foods may be playing a role in these positive food security outcomes. This has implications for conservation policy, suggesting that forests and grasslands in Africa with a nearby human presence are very likely providing wild foods to supplement people's incomes and diets. Restrictive conservation and protected area policies could harm communities' access to these livelihood-supporting resources. Thus, the provisioning ecosystem services offered by these areas could be a justification for supporting conservation efforts and for sustainable use (ICUN Category VI) type protected areas.

## 5 Conclusion

This study shows that communities in areas in Africa with low population densities and high rates of forest and other natural areas are most likely to report collecting wild foods and NTFP. This offers a useful counterpoint to literature drawing only on areas known to have high rates of NTFP collection to examine household characteristics that predict NTFP collection. Furthermore, the observed association between forest cover and wild food collection suggests that wild foods may be playing some role in previously observed associations between forest cover and positive dietary and nutrition outcomes. This has implications for conservation efforts in Africa, suggesting that increased food security via wild food collection can be a justification for conservation, but also that protected areas permitting sustainable use of natural resources will be more beneficial to communities than protected areas that do not give locals access to wild foods or NTFP. Finally, it shows that NTFPs make important contributions to livelihoods in rural landscapes throughout Africa and provides a characterization of landscapes where policy instruments could be targeted to support livelihoods via NTFP.

## Figures and Tables

**Table A.1.1:** Regression for wild foods with geographic variables measured at a 2.5 km buffer around each household. A p-value of <0.001 is indicated with three stars (***), a p-value of <0.01 is indicated with two stars (**), a p-value of <0.05 is indicated with one star (*), and a p-value of <0.1 is indicated with a period (.).

	Estimate	Std. Error	z value	Pr(>|z|)
(Intercept)	−3.43138	1.074157	−3.19449	0.001401**
Area Protected	−2.2174	2.069019	−1.07172	0.283847
Forest Cover	2.294866	0.851461	2.695209	0.007034**
Grassland	0.307016	1.315592	0.233367	0.815476
Head Gender	0.57594	0.453623	1.269646	0.204211
Age	−0.5151	0.890432	−0.57848	0.56294
Years of Schooling	−1.62585	1.136473	−1.43061	0.152543
Literacy	0.145568	0.930138	0.156502	0.875638
Household Size	−0.09113	0.753169	−0.12099	0.903696
Market Distance	−1.61265	2.420055	−0.66637	0.505175
Population Density	−0.2175	1.433043	−0.15177	0.879365
12 – month SPI	0.101208	0.530129	0.190913	0.848594
Critical Food Shortage	−0.02604	0.345313	−0.07542	0.939882
HFIAS	0.500821	1.392471	0.359664	0.719099
Total Ag Production	0.34366	1.776028	0.193499	0.846568
Net Business Income	−0.10878	1.406026	−0.07737	0.938329
Wage Income	−2.82504	2.283327	−1.23725	0.215995
Nonfood Spending	−0.54817	1.86646	−0.2937	0.768989
Food Spending	−0.51271	0.994593	−0.5155	0.606207

**Table A.1.2:** Regression for wild foods with geographic variables measured at a 5 km buffer around each household. A p-value of <0.001 is indicated with three stars (***), a p-value of <0.01 is indicated with two stars (**), a p-value of <0.05 is indicated with one star (*), and a p-value of <0.1 is indicated with a period (.).

	Estimate	Std. Error	z value	Pr(>|z|)
(Intercept)	−3.45593	1.17012	−2.95348	0.003142**
Area Protected	−3.57338	1.962139	−1.82117	0.068582 .
Forest Cover	2.016719	0.932362	2.16302	0.03054*
Grassland	2.072987	1.216874	1.703534	0.088468 .
Head Gender	0.647645	0.451405	1.43473	0.151364
Age	−0.54958	0.890548	−0.61712	0.537153
Years of Schooling	−1.45946	1.128882	−1.29284	0.196067
Literacy	0.138391	0.926983	0.149291	0.881324
Household Size	−0.11711	0.749162	−0.15632	0.875781
Market Distance	−0.19372	2.308423	−0.08392	0.93312
Population Density	0.743553	1.327429	0.560145	0.57538
12 – month SPI	0.074462	0.500711	0.148712	0.881781
Critical Food Shortage	−0.06135	0.347773	−0.17641	0.859972
HFIAS	0.563351	1.387599	0.40599	0.68475
Total Ag Production	0.215317	1.773681	0.121395	0.903378
Net Business Income	−0.08709	1.40453	−0.06201	0.950558
Wage Income	−2.83191	2.306549	−1.22777	0.219534
Nonfood Spending	−0.57118	1.874393	−0.30473	0.760572
Food Spending	−0.60913	0.993946	−0.61284	0.539981

**Table A.1.3:** Regression for wild foods with geographic variables measured at a 10 km buffer around each household. A p-value of <0.001 is indicated with three stars (***), a p-value of <0.01 is indicated with two stars (**), a p-value of <0.05 is indicated with one star (*), and a p-value of <0.1 is indicated with a period (.).

	Estimate	Std. Error	z value	Pr(>|z|)
(Intercept)	−3.36349	1.225662	−2.74422	0.006065**
Area Protected	−0.42656	1.282335	−0.33264	0.739406
Forest Cover	2.17146	1.020222	2.128419	0.033302*
Grassland	2.471235	1.184458	2.086385	0.036944*
Head Gender	0.558173	0.446934	1.248894	0.211704
Age	−0.42809	0.892946	−0.47941	0.631648
Years of Schooling	−1.62095	1.134954	−1.42821	0.153232
Literacy	0.078704	0.918096	0.085725	0.931685
Household Size	−0.06667	0.749821	−0.08891	0.929153
Market Distance	−0.06811	2.45369	−0.02776	0.977854
Population Density	1.05348	1.384996	0.760638	0.446874
12 – month SPI	0.111744	0.491286	0.227453	0.820071
Critical Food Shortage	−0.03709	0.345139	−0.10745	0.914432
HFIAS	0.611404	1.373359	0.445189	0.656183
Total Ag Production	0.313547	1.754836	0.178676	0.858192
Net Business Income	−0.02238	1.408089	−0.01589	0.987318
Wage Income	−2.78523	2.263736	−1.23037	0.21856
Nonfood Spending	−0.62724	1.889475	−0.33197	0.739915
Food Spending	−0.47109	0.995918	−0.47302	0.636199

**Table A.1.4:** Regression for wild foods with geographic variables measured at a 15 km buffer around each household. A p-value of <0.001 is indicated with three stars (***), a p-value of <0.01 is indicated with two stars (**), a p-value of <0.05 is indicated with one star (*), and a p-value of <0.1 is indicated with a period (.).

	Estimate	Std. Error	z value	Pr(>|z|)
(Intercept)	−3.37623	1.1964	−2.82199	0.004773**
Area Protected	−0.78748	1.218518	−0.64626	0.51811
Forest Cover	2.159198	1.107058	1.950392	0.051129 .
Grassland	2.266131	1.285572	1.762742	0.077944 .
Head Gender	0.585464	0.448365	1.305775	0.191629
Age	−0.37245	0.892023	−0.41753	0.676291
Years of Schooling	−1.63467	1.133341	−1.44234	0.149206
Literacy	0.191563	0.915565	0.209229	0.834269
Household Size	−0.07616	0.748265	−0.10179	0.918925
Market Distance	−0.16214	2.51893	−0.06437	0.948676
Population Density	1.114599	1.419501	0.785205	0.432333
12 – month SPI	0.133478	0.482255	0.276778	0.78195
Critical Food Shortage	−0.01297	0.344106	−0.0377	0.969929
HFIAS	0.577112	1.374988	0.419721	0.674689
Total Ag Production	0.403324	1.736556	0.232255	0.81634
Net Business Income	−0.04002	1.408844	−0.0284	0.977339
Wage Income	−2.81653	2.264945	−1.24353	0.213673
Nonfood Spending	−0.6239	1.87312	−0.33308	0.739073
Food Spending	−0.47925	0.991707	−0.48326	0.628912

**Table A.2.1:** Regression for nonfood NTFP with geographic variables measured at a 2.5 km buffer around each household. A p-value of <0.001 is indicated with three stars (***), a p-value of <0.01 is indicated with two stars (**), a p-value of <0.05 is indicated with one star (*), and a p-value of <0.1 is indicated with a period (.).

	Estimate	Std. Error	z value	Pr(>|z|)
(Intercept)	−2.32576	1.000105	−2.32552	0.020044*
Area Protected	1.048476	0.909633	1.152636	0.24906
Forest Cover	0.406747	0.8355	0.48683	0.626379
Grassland	1.02571	0.826946	1.240359	0.214843
Head Gender	0.338702	0.291166	1.163264	0.244722
Age	−0.3606	0.72616	−0.49658	0.619486
Years of Schooling	0.209777	0.83233	0.252036	0.801013
Literacy	−1.2465	0.74164	−1.68074	0.092814 .
Household Size	−0.3466	0.520872	−0.66541	0.505786
Market Distance	−1.05579	1.517361	−0.69581	0.48655
Population Density	−2.98909	1.494319	−2.0003	0.045468*
12 – month SPI	−0.09535	0.453257	−0.21036	0.833387
Critical Food Shortage	0.372993	0.246321	1.514251	0.129962
HFIAS	1.914254	0.980704	1.951919	0.050948 .
Total Ag Production	2.093868	1.474662	1.419897	0.155638
Net Business Income	1.499649	1.471236	1.019312	0.308055
Wage Income	2.030284	1.711244	1.186438	0.235449
Nonfood Spending	1.013976	1.38725	0.730926	0.464825
Food Spending	−0.02732	0.911877	−0.02996	0.976101

**Table A.2.2:** Regression for nonfood NTFP with geographic variables measured at a 5 km buffer around each household. A p-value of <0.001 is indicated with three stars (***), a p-value of <0.01 is indicated with two stars (**), a p-value of <0.05 is indicated with one star (*), and a p-value of <0.1 is indicated with a period (.).

	Estimate	Std. Error	z value	Pr(>|z|)
(Intercept)	−2.10531	1.063251	−1.98007	0.047696*
Area Protected	−0.17409	0.982921	−0.17712	0.859416
Forest Cover	−0.34256	0.962553	−0.35589	0.721922
Grassland	0.7246	0.975684	0.742658	0.457688
Head Gender	0.338835	0.290267	1.167321	0.243081
Age	−0.3592	0.723444	−0.49651	0.619534
Years of Schooling	0.155375	0.828423	0.187555	0.851226
Literacy	−1.2251	0.737777	−1.66053	0.096808 .
Household Size	−0.33946	0.518711	−0.65444	0.51283
Market Distance	−1.17598	1.475353	−0.79708	0.425402
Population Density	−3.07005	1.451267	−2.11543	0.034393*
12 – month SPI	−0.07233	0.443643	−0.16304	0.870484
Critical Food Shortage	0.36907	0.245293	1.504608	0.132425
HFIAS	1.878879	0.981403	1.914483	0.055559 .
Total Ag Production	2.09227	1.479998	1.413697	0.157451
Net Business Income	1.42357	1.457166	0.976944	0.328597
Wage Income	1.996405	1.70616	1.170116	0.241954
Nonfood Spending	0.957612	1.382059	0.692888	0.48838
Food Spending	−0.0373	0.907291	−0.04111	0.967208

**Table A.2.3:** Regression for nonfood NTFP with geographic variables measured at a 10 km buffer around each household. A p-value of <0.001 is indicated with three stars (***), a p-value of <0.01 is indicated with two stars (**), a p-value of <0.05 is indicated with one star (*), and a p-value of <0.1 is indicated with a period (.).

	Estimate	Std. Error	z value	Pr(>|z|)
(Intercept)	−1.96171	1.106792	−1.77242	0.076324 .
Area Protected	−1.7841	0.952647	−1.87279	0.061098 .
Forest Cover	−0.83523	0.970466	−0.86065	0.389431
Grassland	0.504079	1.111416	0.453547	0.650155
Head Gender	0.359377	0.29044	1.237352	0.215957
Age	−0.42715	0.722858	−0.59092	0.554572
Years of Schooling	0.173456	0.82539	0.21015	0.83355
Literacy	−1.23818	0.737157	−1.67967	0.093021 .
Household Size	−0.36074	0.51724	−0.69743	0.485532
Market Distance	−1.4531	1.37173	−1.05932	0.289454
Population Density	−3.11725	1.406617	−2.21613	0.026682*
12 – month SPI	−0.108	0.420577	−0.25679	0.797339
Critical Food Shortage	0.368063	0.245247	1.500784	0.133411
HFIAS	1.77515	0.989271	1.794403	0.072749 .
Total Ag Production	2.08933	1.488202	1.403929	0.16034
Net Business Income	1.31846	1.439155	0.916135	0.359596
Wage Income	1.912044	1.694815	1.128173	0.259247
Nonfood Spending	0.926001	1.381948	0.670069	0.502814
Food Spending	−0.04431	0.903015	−0.04907	0.960861

**Table A.2.4:** Regression for nonfood NTFP with geographic variables measured at a 15 km buffer around each household. A p-value of <0.001 is indicated with three stars (***), a p-value of <0.01 is indicated with two stars (**), a p-value of <0.05 is indicated with one star (*), and a p-value of <0.1 is indicated with a period (.).

	Estimate	Std. Error	z value	Pr(>|z|)
(Intercept)	−2.3202	1.150867	−2.01605	0.043795*
Area Protected	−2.12371	0.863384	−2.45976	0.013903*
Forest Cover	−0.94717	1.086216	−0.87199	0.383214
Grassland	0.957735	1.181663	0.810497	0.417654
Head Gender	0.371083	0.290894	1.275665	0.202074
Age	−0.48117	0.72305	−0.66548	0.505746
Years of Schooling	0.183229	0.823561	0.222484	0.823937
Literacy	−1.22596	0.737732	−1.6618	0.096553 .
Household Size	−0.36005	0.517268	−0.69606	0.486389
Market Distance	−1.78771	1.300162	−1.37499	0.169134
Population Density	−3.07212	1.361984	−2.25562	0.024094*
12 – month SPI	−0.07209	0.401516	−0.17955	0.857503
Critical Food Shortage	0.369701	0.245481	1.506023	0.132061
HFIAS	1.816762	0.990745	1.833734	0.066694 .
Total Ag Production	2.129462	1.489305	1.429836	0.152764
Net Business Income	1.33032	1.441326	0.922983	0.356016
Wage Income	1.934614	1.707304	1.133139	0.257156
Nonfood Spending	0.888392	1.390335	0.638977	0.522838
Food Spending	−0.02371	0.904276	−0.02623	0.979078

**Table B.1:** Summary statistics for Ghana. Note: For some surveys, only the month of interview was recorded, so we were unable to calculate a time span in days.

Landscape		Pwalagu	Diari	Atebubu	Kuntanse	Mpataba	Nsobri	All
Fraction of Land Area within 7.5 km Falling Within A Protected Area	Min	0	0	0	0	0	0	0
	Max	0	0	0	0	0.000933	0	0.000933
	Mean	0	0	0	0	5.88E-05	0	9.59E-06
Fraction of Land Area within 7.5 km that is Forest	Min	0.193353	0.054212	0.805812	0	0.012082	0	0
	Max	0.323074	0.176881	0.948859	0.001766	0.056724	0.000535	0.948859
	Mean	0.268184	0.101059	0.926847	0.000205	0.020944	4.07E-05	0.213127
Fraction of Land Area within 7.5 km that is Shrubland, Grassland or Herbaceous	Min	0.236212	0.163636	0	0	0	0	0
	Max	0.36666	0.418738	0	0	0.004899	5.12E-05	0.418738
	Mean	0.279614	0.274979	0	0	0.000816	1.8E-06	0.086073
Average Age of Individuals Within a Household	Min	13.75	14.16667	13.16667	12.71429	19.4	12.42857	12.42857
	Max	65	45	82	71	53	79.66667	82
	Mean	28.34256	21.19691	33.63778	34.4699	29.8013	33.27661	30.37991
Average Years of Schooling Within a Household	Min	0	0	0	3	1.8	0	0
	Max	8.333333	9	13.5	13	9	12.4	13.5
	Mean	3.260423	2.667445	4.195503	7.584606	5.708034	4.659085	4.695319
Household Size	Min	1	3	1	1	2	1	1
	Max	14	18	14	10	11	12	18
	Mean	6.366667	7.37037	4.2	4.862069	5.6	5.447368	5.608696
Fraction of Household Members That Are Literate	Min	0	0	0	0	0	0	0
	Max	0.8	0.714286	1	1	1	1	1
	Mean	0.320212	0.270415	0.379286	0.547906	0.577165	0.483991	0.434141
12 - Month Standardized Precipitation Index	Min	−1.3498	−1.20088	−0.92656	0.062435	0.48072	−2.61529	−2.61529
	Max	−1.3498	−1.20088	−0.92656	0.062435	0.48072	−0.80278	0.48072
	Mean	−1.3498	−1.20088	−0.92656	0.062435	0.48072	−1.99522	−0.8712
Household Food Insecurity and Access Score (HFIAS)	Min	0	0	0	0	0	0	0
	Max	18	7	0	10	3	20	20
	Mean	0.833333	0.259259	0	1.206897	0.1	1.736842	0.73913
Total Agricultural Production Value (In 2015 USD)	Min	40.1141	67.78244	88.15159	0	0	0	0
	Max	4376.083	11,693.34	9998.421	6734.545	51,428.37	23,710.27	51,428.37
	Mean	960.3247	2133.721	2401.148	1869.752	5994.188	3190.848	2792.148
Net Income from Businesses (in 2015 USD)	Min	0	0	0	0	0	0	0
	Max	235.2145	75.31382	35.83206	266.2295	331.0488	570.1325	570.1325
	Mean	26.49961	7.419806	3.857918	16.21475	37.90509	39.47235	22.92604
Income from Wages (in 2015 USD)	Min	0	0	0	0	0	0	0
	Max	3993.176	2530.544	687.9755	12,146.72	4767.103	3450.611	12,146.72
	Mean	194.371	169.4561	22.93252	1367.557	221.0066	573.4063	430.2893
Annual Nonfood Spending (in 2015 USD)	Min	121.2737	126.3118	13.55455	22.19622	122.2563	485.7528	13.55455
	Max	29,903.84	12,744.96	14,855.02	64,703.86	18,311.25	72,450.52	72,450.52
	Mean	4490.666	2385.599	2292.433	7564.672	3945.667	9021.089	5154.625
Annual Food Spending (in 2015 USD)	Min	306.1267	569.3951	112.097	277.6241	552.3484	345.0422	112.097
	Max	6702.464	5703.768	5343.291	4853.217	3973.456	7551.115	7551.115
	Mean	1402.669	1708.403	983.2154	1603.1	1736.042	2131.718	1615.651
Household Interview Date	Latest	2/2015	11/2014	9/2014	8/2014	3/2015	7/3/2014	3/19/2015
	Earliest	2/2015	11/2014	9/2014	8/2014	3/2015	1/1/2014	1/1/2014
	Span	–	–	–	–	–	183 days	442 days
Fraction of Households that Collect Wild Foods		0.666667	0.518519	0.533333	0.034483	0.133333	0.5	0.402174
Fraction of Households that Collect Nonfood NTFP		0.5	0.62963	0.733333	0.724138	0.233333	0.789474	0.608696
Fraction of Households with a Male Head		0.933333	1	0.8	0.862069	0.933333	0.947368	0.913044
Fraction of Households Who Experienced a Critical Food Shortage in the Past Year		0.133333	0.148148	0.066667	0.172414	0	0.315789	0.146739
Market Distance - Distance to a town with >50 k people, in hours		1.699824	2.342495	4.574964	1.076525	3.533333	1.129825	2.345889
Population Density (People per 100 sq. km)		171.1808	112.4805	310.2312	2332.233	240.7951	4310.007	1391.947
Total Number of Households		30	27	30	29	30	38	184

**Table B.2:** Summary statistics for Rwanda.

Landscape		Nyungwe	Volcanoes	Akagera	Gishwati	Muhanga-Kamonyi	Bugesera	All
Fraction of Land Area within 7.5 km Falling Within A Protected Area	Min	0.041772	0.001746	0.031834	0.059883	0	0	0
	Max	0.371235	0.311612	0.285122	0.083033	0	0	0.371235
	Mean	0.188161	0.166798	0.129716	0.075677	0	0	0.093392
Fraction of Land Area within 7.5 km that is Forest	Min	0.169951	0.308095	0.031608	0.269233	0.007975	0.044293	0.007975
	Max	0.358821	0.5637	0.092278	0.443543	0.292354	0.109712	0.5637
	Mean	0.266655	0.471818	0.07027	0.356573	0.048748	0.072125	0.214365
Fraction of Land Area within 7.5 km that is Shrubland, Grassland or Herbaceous	Min	0	8.57E-06	0.307682	0	0	0	0
	Max	0	0.002732	0.595587	0	0.006555	0.00983	0.595587
	Mean	0	0.001538	0.469965	0	0.003368	0.002961	0.079639
Average Age of Individuals Within a Household	Min	14.125	15	12.16667	12.42857	16	7.2	7.2
	Max	48.5	40.16667	56.33333	47.5	51	67.5	67.5
	Mean	24.50788	24.53778	23.84317	24.82517	25.97556	23.92906	24.60311
Average Years of Schooling Within a Household	Min	0	1	0.5	0	0	1	0
	Max	6.833333	7.5	11	8	8	7.8	11
	Mean	2.433963	3.623571	4.053981	3.155026	3.739221	2.81496	3.303454
Household Size	Min	2	2	3	2	2	2	2
	Max	11	10	9	9	11	10	11
	Mean	5.666667	4.866667	5.233333	5.7	4.333333	5.166667	5.161111
Fraction of Household Members That Are Literate	Min	0	0	0	0	0	0.2	0
	Max	1	1	1	1	1	1	1
	Mean	0.408354	0.555397	0.589749	0.566257	0.551039	0.539749	0.535091
12 - Month Standardized Precipitation Index	Min	−0.49094	0.670096	−0.30882	0.174328	−0.66107	−0.32331	−0.66107
	Max	0.822075	1.689018	−0.30882	0.542929	−0.50796	−0.32331	1.689018
	Mean	0.186615	1.506217	−0.30882	0.334055	−0.59983	−0.32331	0.132488
Household Food Insecurity and Access Score (HFIAS)	Min	0	0	0	0	0	0	0
	Max	31	21	31	29	41	28	41
	Mean	13.03333	5	11.53333	10	7.233333	15.4	10.36667
Total Agricultural Production Value (In 2015 USD)	Min	0	19.47508	19.21156	11.38609	0	28.69262	0
	Max	1069.585	3960.784	1228.555	3162.582	3850.835	2448.631	3960.784
	Mean	391.124	775.6163	409.8512	620.9853	677.1648	371.4404	541.0303
Net Income from Businesses (in 2015 USD)	Min	0	0	0	0	0	0	0
	Max	49.9632	16.4436	32.36082	24.7695	231.6818	8.098217	231.6818
	Mean	5.374029	2.13723	1.078694	0.82565	10.91561	0.344536	3.445958
Income from Wages (in 2015 USD)	Min	0	0	0	0	0	0	0
	Max	1879.876	9341.697	2509.466	1291.553	4305.041	1575.297	9341.697
	Mean	406.035	991.0128	544.7325	411.6677	607.4689	347.0989	551.3359
Annual Nonfood Spending (in 2015 USD)	Min	36.51473	260.1121	40.52241	35.53988	2.69364	33.35138	2.69364
	Max	55,770.44	41,857.62	18,144.19	22,548.91	110,744.9	5818.593	110,744.9
	Mean	3884.277	8959.775	3586.879	4101.092	6679.994	1503.204	4785.87
Annual Food Spending (in 2015 USD)	Min	8.634367	16.93187	92.20586	43.00855	136.5502	97.11544	8.634367
	Max	2268.887	1414.799	1248.97	2017.62	2600.102	899.3734	2600.102
	Mean	495.5233	505.9889	522.9997	655.9053	576.7199	359.2694	519.4011
Household Interview Date	Latest	1/16/2016	5/28/2016	7/29/2016	2/17/2016	11/14/2015	8/25/2016	8/25/2016
	Earliest	10/20/2015	2/29/2016	7/12/2016	1/18/2016	10/1/2015	8/9/2016	10/1/2015
	Span	88 days	89 days	17 days	30 days	44 days	16 days	329 days
Fraction of Households that Collect Wild Foods		0	0	0.033333	0	0	0	0.005556
Fraction of Households that Collect Nonfood NTFP		0	0	0	0.033333	0.133333	0	0.027778
Fraction of Households with a Male Head		0.8	0.666667	0.7	0.8	0.666667	0.7	0.722222
Fraction of Households Who Experienced a Critical Food Shortage in the Past Year		0.633333	0.8	0.9	0.833333	0.633333	0.933333	0.788889
Market Distance - Distance to a town with >50 k people, in hours		5.051842	3.175238	6.320992	5.187238	2.74076	3.448276	4.320724
Population Density (People per 100 sq. km)		4159.321	11,447.38	2502.143	11,163.86	9695.928	5402.908	7395.256
Total Number of Households		30	30	30	30	30	30	180

**Table B.3:** Summary statistics for Uganda.

Landscape		Yumbe	Bududa	Butambala	Kisoro	Masindi	Otuke	All
Fraction of Land Area within 7.5 km Falling Within A Protected Area	Min	0	0.062862	0	0	0.070638	0	0
	Max	0	0.343483	0	0	0.585955	0	0.585955
	Mean	0	0.182292	0	0	0.345858	0	0.089014
Fraction of Land Area within 7.5 km that is Forest	Min	0.623982	0.012726	0	0.177784	0.057168	0.490966	0
	Max	0.945402	0.054838	0.037061	0.306267	0.229435	0.726289	0.945402
	Mean	0.752456	0.031018	0.003829	0.256285	0.145689	0.645033	0.307692
Fraction of Land Area within 7.5 km that is Shrubland, Grassland or Herbaceous	Min	0.003272	0	0.000461	0.001093	0.043642	0.192248	0
	Max	0.035043	0	0.025799	0.002732	0.167627	0.489238	0.489238
	Mean	0.023052	0	0.002401	0.00187	0.118497	0.25476	0.06749
Average Age of Individuals Within a Household	Min	9.5	11.5	11.85714	12.33333	12	13	9.5
	Max	44.33333	79	67.5	42	36	38.16667	79
	Mean	21.29366	27.18761	27.94485	23.52429	20.28477	22.48562	23.76492
Average Years of Schooling Within a Household	Min	0.8	0	1.625	0	1.428571	1.75	0
	Max	6.071429	11	8.5	8.666667	9	10	11
	Mean	2.425978	4.114387	4.318733	4.078695	4.155225	4.351282	3.90411
Household Size	Min	3	2	1	2	2	2	1
	Max	16	11	10	10	12	13	16
	Mean	7.833333	6.3	6.137931	5.517241	7.266667	5.666667	6.460674
Fraction of Household Members That Are Literate	Min	0	0	0	0	0.111111	0	0
	Max	0.642857	1	1	0.833333	1	0.714286	1
	Mean	0.211081	0.311811	0.514299	0.398495	0.433909	0.366441	0.371732
12 - Month Standardized Precipitation Index	Min	0.302376	0.160638	−1.04069	0.53064	−0.09708	0.236382	−1.04069
	Max	0.536668	0.382087	−0.65505	0.53064	0.333761	0.586303	0.586303
	Mean	0.452464	0.190165	−0.84122	0.53064	−0.054	0.283039	0.096311
Household Food Insecurity and Access Score (HFIAS)	Min	0	0	0	0	0	0	0
	Max	21	9	10	7	6	7	21
	Mean	4.266667	2.333333	1.206897	2.517241	0.466667	2.233333	2.174157
Total Agricultural Production Value (In 2015 USD)	Min	53.07215	28.30589	89.07412	16.08777	49.99008	7.615806	7.615806
	Max	2623.75	2456.025	7462.278	3268.274	6968.8	1906.934	7462.278
	Mean	382.1604	869.2994	980.3162	286.0518	786.3312	332.1614	605.7486
Net Income from Businesses (in 2015 USD)	Min	0	0	0	0	0	0	0
	Max	66.27307	41.04682	42.55558	36.70621	51.40312	6.697344	66.27307
	Mean	7.566244	3.245201	7.907065	2.241356	3.884296	0.669332	4.243014
Income from Wages (in 2015 USD)	Min	0	0	0	0	0	0	0
	Max	42,433.04	1970.247	4659.836	15,717.89	3127.023	1826.787	42,433.04
	Mean	2624.254	300.7685	467.2139	1196.457	345.7786	287.2102	870.7123
Annual Nonfood Spending (in 2015 USD)	Min	124.234	14.17361	223.3138	144.0365	552.8831	170.8051	14.17361
	Max	40,078.14	30,643.67	73,993.52	24,022.45	12,836.18	16,765.75	73,993.52
	Mean	6367.771	5109.872	6578.37	5664.827	3961.572	3136.196	5125.365
Annual Food Spending (in 2015 USD)	Min	21.28602	28.6933	137.8407	13.42864	135.6968	50.63393	13.42864
	Max	2562.813	1937.329	1579.07	2616.339	2417.572	1562.659	2616.339
	Mean	860.5339	784.7564	580.249	637.3069	732.8126	421.41	670.1939
Household Interview Date	Latest	7/19/2016	5/14/2015	3/27/2015	3/25/2016	10/4/2015	10/4/2015	7/19/2016
	Earliest	5/26/2016	4/28/2015	2/2/2015	3/9/2016	8/14/2015	9/16/2015	2/2/2015
	Span	54 days	16 days	53 days	16 days	51 days	18 days	533 days
Fraction of Households that Collect Wild Foods		0.8	0.233333	0.068966	0.103448	0.033333	0.266667	0.252809
Fraction of Households that Collect Nonfood NTFP		0.4	0	0.344828	0.413793	0.4	0.7	0.376404
Fraction of Households with a Male Head		0.933333	0.833333	0.862069	0.862069	0.9	0.833333	0.870787
Fraction of Households Who Experienced a Critical Food Shortage in the Past Year		0.8	0.8	0.344828	0.862069	0.5	0.933333	0.707865
Market Distance - Distance to a town with >50 k people, in hours		6.887001	3.335377	1.864807	4.753443	4.546038	2.632934	4.011066
Population Density (People per 100 sq. km)		2541.423	13,202.24	2439.872	5901.509	1695.123	893.9224	4448.771
Total Number of Households		30	30	29	29	30	30	178

**Table B.4:** Summary statistics for Tanzania.

Landscape		Sumbawanga	Mufindi	Ludewa	Kilolo	Kilombero	Mbarali	Rufiji	All
Fraction of Land Area within 7.5 km Falling Within A Protected Area	Min	0	0	0	0	0	0	0	0
	Max	0	0	0	0.177275	0.445712	0	0	0.445712
	Mean	0	0	0	0.024795	0.082287	0	0	0.014977
Fraction of Land Area within 7.5 km that is Forest	Min	0.15751534	0.065715	0.80733	0.024597	0.04117	0.001081	0.477349	0.001081
	Max	0.2983656	0.297375	0.900286	0.641851	0.632093	0.008013	0.861439	0.900286
	Mean	0.240994	0.179029	0.84343	0.200385	0.276262	0.004755	0.637894	0.340699
Fraction of Land Area within 7.5 km that is Shrubland, Grassland or Herbaceous	Min	0.1588905	0.060831	0.018655	0.292981	0.03596	0.475732	0.030417	0.018655
	Max	0.5100406	0.197286	0.049237	0.79577	0.40498	0.835244	0.036328	0.835244
	Mean	0.3396921	0.115382	0.031382	0.654383	0.193417	0.656782	0.033624	0.289696
Average Age of Individuals Within a Household	Min	11.2	13.8	13.25	14	13	12.57143	11.75	11.2
	Max	64	53.66667	69	43.66667	51	41.6	56.5	69
	Mean	25.86128	24.82095	32.3781	25.20139	25.59893	22.15307	26.9833	26.14503
Average Years of Schooling Within a Household	Min	0	1.166667	0	0	0.6	0	0	0
	Max	11.5	6.5	8.833333	9.6	8	9	8	11.5
	Mean	3.917462	3.334061	4.777937	4.596349	4.160345	3.264371	3.160114	3.885927
Household Size	Min	2	3	1	2	1	2	2	1
	Max	11	9	7	8	8	17	11	17
	Mean	5.566667	5.333333	3.633333	4.8	5.068966	7.2	5.066667	5.239234
Fraction of Household Members That Are Literate	Min	0	0.333333	0	0	0.2	0	0	0
	Max	1	1	1	1	1	1	1	1
	Mean	0.6289442	0.628704	0.754921	0.718373	0.756363	0.531253	0.524444	0.648487
12 - Month Standardized Precipitation Index	Min	−0.9734436	0.078587	−1.28784	−0.87135	0.65233	1.583463	−0.1264	−1.28784
	Max	0.04681146	0.578645	−1.28784	−0.87135	0.747955	1.62415	−0.10705	1.62415
	Mean	−0.2932736	0.295279	−1.28784	−0.87135	0.744657	1.605163	−0.11286	0.007889
Household Food Insecurity and Access Score (HFIAS)	Min	0	0	0	0	0	0	0	0
	Max	11	18	0	23	15	7	12	23
	Mean	0.9666667	2.766667	0	5.833333	1.965517	0.5	2.3	2.047847
Total Agricultural Production Value (In 2015 USD)	Min	42.34982	23.83796	32.28449	12.06405	0	44.01729	41.33025	0
	Max	12,599.342	1854.285	3198.63	2685.785	6759.372	42,367.23	1057.469	42,367.23
	Mean	874.9965	320.8736	602.4073	721.9336	1314.262	4150.735	341.4954	1188.932
Net Income from Businesses (in 2015 USD)	Min	0	0	0	0	0	0	0	0
	Max	10.637622	150.7105	40.35562	150.6741	0	14.64404	14.38232	150.7105
	Mean	0.3545874	17.25559	3.406543	8.537746	0	1.463967	1.235752	4.629787
Income from Wages (in 2015 USD)	Min	0	0	0	0	0	0	0	0
	Max	6414.1091	2754.221	3508.519	8937.363	9029.513	18,744.03	2399.793	18,744.03
	Mean	448.49825	301.1677	550.27	872.1827	2148.843	2073.445	357.353	958.8706
Annual Nonfood Spending (in 2015 USD)	Min	30.504446	11.16313	19.68934	8.143606	30.19883	73.51915	18.08716	8.143606
	Max	13,347.379	7990.349	9405.639	8060.684	7579.771	58,739.48	5413.313	58,739.48
	Mean	1759.832	1123.245	1556.949	1344.425	962.6138	3791.743	802.4205	1623.322
Annual Food Spending (in 2015 USD)	Min	25.81877	47.0624	135.7203	276.9449	97.71665	62.99206	143.1768	25.81877
	Max	1621.7644	1759.804	2389.955	2231.272	2445.626	4094.484	2108.318	4094.484
	Mean	432.1263	628.0327	738.7798	1068.076	967.8803	1181.718	876.4258	841.2597
Household Interview Date	Latest	12/5/2014	12/13/2013	3/29/2015	10/26/2013	3/1/2014	10/10/2014	6/8/2015	6/8/2015
	Earliest	11/17/2014	11/16/2013	3/9/2015	10/1/2013	2/1/2014	9/22/2014	5/16/2015	10/1/2013
	Span	18 days	27 days	20 days	25 days	28 days	18 days	23 days	615 days
Fraction of Households that Collect Wild Foods		0.06666667	0	0	0.066667	0	0	0.066667	0.028708
Fraction of Households that Collect Nonfood NTFP		0.43333333	0.533333	0.333333	0.6	0.310345	0.233333	0.9	0.478469
Fraction of Households with a Male Head		1	0.666667	0.8	0.866667	0.862069	0.8	0.9	0.842105
Fraction of Households Who Experienced a Critical Food Shortage in the Past Year		0.2	0.6	0.066667	0.633333	0.482759	0.4	0.466667	0.406699
Market Distance - Distance to a town with >50 k people, in hours		5.763255	4.971429	16.48692	4.270021	3.992333	6.206288	9.042837	7.263159
Population Density (People per 100 sq. km)		1132.09018	1051.845	641.4672	59.27397	868.2811	1987.59	108.04	835.3557
Total Number of Households		30	30	30	30	29	30	30	209
